# Genetic testing for familial hyperparathyroidism: clinical-genetic profile in a Mediterranean cohort

**DOI:** 10.3389/fendo.2023.1244361

**Published:** 2023-09-21

**Authors:** Isabel Mazarico-Altisent, Ismael Capel, Neus Baena, Maria Rosa Bella-Cueto, Santi Barcons, Xavier Guirao, Rocío Pareja, Andreea Muntean, Valeria Arsentales, Assumpta Caixàs, Mercedes Rigla

**Affiliations:** ^1^ Endocrinology and Nutrition Department, Parc Taulí University Hospital, Institut d’Investigació i Innovació Parc Taulí (I3PT), Medicine Department, Universitat Autònoma de Barcelona, Sabadell, Barcelona, Spain; ^2^ Genetic Department, Parc Taulí University Hospital, Institut d’Investigació i Innovació Parc Taulí (I3PT), Medicine Department, Universitat Autònoma de Barcelona, Sabadell, Barcelona, Spain; ^3^ Pathology Department, Parc Taulí University Hospital, Institut d’Investigació i Innovació Parc Taulí (I3PT), Medicine Department, Universitat Autònoma de Barcelona, Sabadell, Barcelona, Spain; ^4^ Surgery Department, Parc Taulí University Hospital, Institut d’Investigació i Innovació Parc Taulí (I3PT), Medicine Department, Universitat Autònoma de Barcelona, Sabadell, Barcelona, Spain

**Keywords:** parathyroid, primary hyperparathyroidism, multiple endocrine neoplasia type 1, multiple endocrine neoplasia type 4, cyclin-dependent kinase inhibitors, familial hyperparathyroidism, familial hypocalciuric hypercalcemia

## Abstract

**Background:**

Approximately 10% of primary hyperparathyroidism cases are hereditary, due to germline mutations in certain genes. Although clinically relevant, a systematized genetic diagnosis is missing due to a lack of firm evidence regarding individuals to test and which genes to evaluate.

**Methods:**

A customized gene panel (*AIP*, *AP2S1*, *CASR*, *CDC73*, *CDKN1A*, *CDKN1B*, *CDKN2B*, *CDKN2C*, *GCM2*, *GNA11*, *MEN1*, *PTH*, *RET*, and *TRPV6*) was performed in 40 patients from the Mediterranean area with suspected familial hyperparathyroidism (≤45 years of age, family history, high-risk histology, associated tumour, multiglandular disease, or recurrent hyperparathyroidism). We aimed to determine the prevalence of germline variants in these patients, to clinically characterize the probands and their relatives, and to compare disease severity in carriers versus those with a negative genetic test.

**Results:**

Germline variants were observed in 9/40 patients (22.5%): 2 previously unknown pathogenic/likely pathogenic variants of *CDKN1B* (related to MEN4), 1 novel variant of uncertain significance of *CDKN2C*, 4 variants of *CASR* (3 pathogenic/likely pathogenic variants and 1 variant of uncertain significance), and 2 novel variants of uncertain significance of *TRPV6*. Familial segregation studies allowed diagnosis and early treatment of PHPT in first-degree relatives of probands.

**Conclusion:**

The observed prevalence of germline variants in the Mediterranean cohort under study was remarkable and slightly higher than that seen in other populations. Genetic screening for suspected familial hyperparathyroidism allows the early diagnosis and treatment of PHPT and other related comorbidities. We recommend genetic testing for patients with primary hyperparathyroidism who present with high-risk features.

## Introduction

Primary hyperparathyroidism (PHPT) is a common endocrine disorder, with an overall incidence of 13-78 per 100,000 person-years in the world (1). Owing to more widespread screening for hypercalcemia, this frequency has risen in recent years (1–3). The prevalence rises with age and typically exhibits a female predominance (1). If left untreated, PHPT can cause hypercalcaemic syndrome, with increased mortality, muscle weakness, nephrocalcinosis, renal stones, osteoporosis, bone fractures, and heart failure among others (1). Up to 10% of all PHPT cases are due to germline variants in recurrent genes (4, 5), a situation known as familial primary hyperparathyroidism (FHPT) (6). This prevalence increases to 15-26% in patients with risk features (7). However, it appears that FHPT is underdiagnosed and underreported in the literature, likely due to the limited number of genetic studies performed in clinical practice and an insufficient understanding of these conditions.

FHPT may occur either alone (without other related tumours) or as part of a syndrome, such as multiple endocrine neoplasia (MEN) types 1, 2A, and 4, and hyperparathyroidism-jaw tumour syndrome. MEN1 is caused by inactivating germline variants of the *MEN1* tumour suppressor gene that produce PHPT, gastro-entero-pancreatic neuroendocrine tumours, pituitary adenomas, and other tumours, such as bronchial/thymic carcinoids or lipomas (8). MEN2A is caused by activating variants in the proto-oncogene *RET* and typically presents as medullary thyroid carcinoma, pheochromocytoma, and parathyroid tumours (20-30%) with a lower penetrance (9). Patients with MEN4 express MEN1-like milder phenotypes due to *CDKN1B* germline variants and, less frequently, other non-endocrine neoplasms such as breast cancer, prostate cancer, colon cancer, papillary thyroid carcinoma, angiomyolipoma, and meningioma, although only approximately 100 cases have been reported to date (10, 11). Variants in other cyclin-dependent kinase inhibitors (*CDKIs*), such as *CDKN1A*, *CDKN2B*, and *CDKN2C*, have been described in patients with the MEN1 phenotype or suspected FHPT, despite limited data (11, 12). Recently, germline variants of the *MAX* tumour suppressor gene have been associated with a new entity, MEN5. It is thought to cause pheochromocytomas and paragangliomas and, possibly, parathyroid adenomas and functional pituitary adenomas (13, 14). Hyperparathyroidism-jaw tumour syndrome is caused by germline inactivating variants of the *CDC73* (also called *HRPT2*) tumour suppressor gene. These mutations can result in aggressive forms of PHPT (parathyroid carcinoma, atypical parathyroid adenoma), fibroosseous jaw tumours, and renal and uterine tumours (15, 16).

FHPT may also present as non-syndromic PHPT, which includes familial hypocalciuric hypercalcemia (FHH) forms (caused by variants of *CASR*, *GNA11*, or *AP2S1*), neonatal severe hyperparathyroidism, and other forms caused by several genes classified as familial isolated hyperparathyroidism (FIHP). FIHP is diagnosed by exclusion because the genetic cause is unknown in most cases (17–19). *GCM2* proto-oncogene variants are present in about 18% of these cases, while about 30% involve mutations in *MEN1* or *CDC73* with incomplete expression or loss-of-function variants of the *CASR* gene (1, 4, 20–22). Other genes related to familial hyperparathyroidism are *AIP* (23) and *TRPV6* (24). In recent years, the number of genes potentially involved in parathyroid tumorigenesis is constantly expanding (25).

Most authors propose performing genetic testing in patients with PHPT who present some risk features suspicious of FHPT: diagnosis under 30-45 years, high-risk parathyroid histology (multiglandular disease (MGD), parathyroid carcinoma, atypical adenoma, or cystic adenoma), carrying tumours related to syndromic FHPT, family history of PHPT, or a calcium creatinine clearance ratio (CCCR) <0,02 (7). However, consensual systematized genetic screening for FHPT has not been widely implemented because of the lack of firm evidence on which individuals should be tested and which genes should be evaluated. Few studies on the systematic search for germline variants in patients with suspected FHPT have been conducted, and those that have been done have shown mixed results. In addition, these studies were performed in various clinical scenarios, with heterogeneous genetic testing methodologies and various genes assessed (7, 26, 27). To our knowledge, none of these were carried out among the Spanish population, and only a few involved people living in the Mediterranean area.

Genetic testing is of considerable interest to patients with PHPT of a presumed familial origin. This allows for: 1) Predicting which patients are at risk of developing other neoplasms (ex. neuroendocrine tumours in MEN1 or MEN4). 2) Planning the best management of PHPT for each patient and aid in surgical necessity and planning. 3) Guiding surveillance for other clinical features associated with a given genetic anomaly. 4) Carrying out cascade testing of asymptomatic relatives, which allows for early diagnosis of potentially severe syndromes and reduces healthcare burden and psychological stress in mutation-negative relatives. 5) Offering optimal genetic counselling if needed via prenatal/preimplantation diagnosis (1, 3, 7, 11, 15).

The aim of this study was to perform genetic screening, at our hospital, of patients with PHPT who presented some risk features, in order to: 1) Determine the prevalence of germline mutations in the analysed genes in our cohort. 2) Clinically characterize carriers of variants and their first-degree relatives. 3) Compare disease severity in carriers of variants with respect to those without a genetic alteration found and compare the prevalence of germline variants in our population with those in other cohorts.

## Materials and methods

### Clinical data collection

An ambispective observational study was conducted. We collected data from patients diagnosed with PHPT who also exhibited some risk factors for FHPT. The diagnosis of PHPT was established by inappropriately non-suppressed or high parathyroid hormone levels (PTH) (reference value [RV] 12.32-41.99 pmol/L) with normal or elevated albumin-corrected serum calcium levels (RV 2.1-2.55 mmol/L). Patients were included in the study when at least one inclusion criterion was met: 1) Age at onset of PHPT ≤ 45 years. 2) High-risk parathyroid histology (parathyroid carcinoma, atypical parathyroid adenoma, cystic parathyroid adenoma, or biopsy-documented MGD defined as having two or more glands affected by either adenomas or hyperplasia). 3) Recurrent or persistent PHPT (established by presenting corrected serum calcium levels ≥ 2.62 mmol/L three months after parathyroid surgery). 4) Family history of PHPT in at least one first-degree relative. 5) Other tumour/s related to syndromic FHPT (gastro-entero-pancreatic neuroendocrine tumours, pituitary adenoma, jaw tumour, renal or uterine tumour, medullary thyroid carcinoma, breast tumour, malignant hemopathy, or others). Having a known mutation in any of the genes under study, as well as having a first-degree relative with a known mutation in any of them, were the only exclusion criteria.

**Figure 1:**
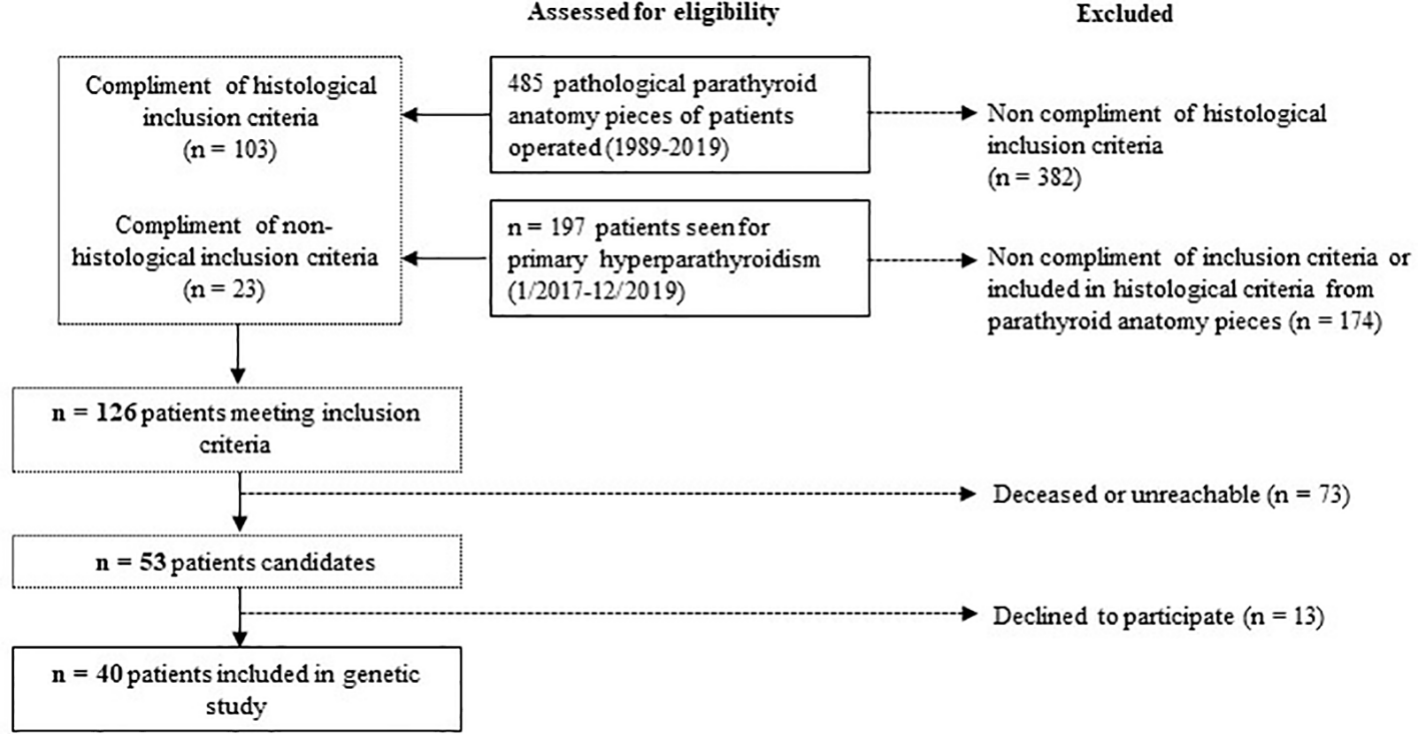
Study design.

We retrospectively reviewed the medical records, from pathologic data base, of all patients who underwent surgery for hyperparathyroidism between 1989-2019 at the Parc Taulí University Hospital. In total, 103/485 patients met the histological inclusion criteria. We also examined all patients referred to the Endocrinology and Nutrition Department of our hospital for PHPT from January 2017 to December 2019 to include patients with PHPT who had not yet undergone surgery. Of them, 23/197 met inclusion criteria. In total, 126 patients met at least one of the inclusion criteria. Seventy-three patients were deceased or unreachable and 13 declined to participate. In the end, 40 index cases were included. The study design is illustrated in **Figure 1**.

Patients who met the inclusion criteria and agreed to participate were interviewed to obtain detailed medical and family histories between February and July 2020. Baseline data were recorded for each patient, including age at presentation, sex, initial clinical presentation, preoperative and postoperative biochemical and hormonal findings (PTH, calcium, phosphate, and 25-hydroxy vitamin D levels), and calcium creatinine clearance ratio (CCCR, calculated using a 24-hour urinary calcium collection and one blood sample) at diagnosis when available. We reviewed the preoperative imaging results of the parathyroid glands, type of surgery performed, surgical findings, histopathology, postoperative data, and follow-up outcomes.

Written informed consent was obtained from all patients, and the study was approved by the institutional Ethics Committee of Institut d’Investigació i Innovació Parc Taulí I3PT.

### Gene panel sequencing and variant interpretation

DNA was extracted from blood and other tissue samples using the Gentra Puregene DNA reagent (Qiagen, Valencia, CA). Genetic screening for FHPT was performed using a customized gene panel that included *AIP*, *AP2S1*, *CASR*, *CDC73*, *CDKN1A*, *CDKN1B*, *CDKN2B*, *CDKN2C*, *GCM2*, *GNA11*, *MEN1*, *PTH*, *RET*, and *TRPV6*, through both sequence analysis and copy number variation (CNV) analysis. This gene panel was selected based on recent data and previous studies (15, 28, 29).

In the analysis, the mean sequencing depth was > 150 times, and > 99% of the target nucleotides were covered with > 20× sequencing depth for all assays. The target nucleotides included all protein-coding exons of the genes in the panels in addition to 20 base pairs inside each intron/exon boundary. The panel was also customized by adding oligonucleotides that targeted deep intronic variants (≥ 20 base pairs from the intron/exon boundary) and noncoding variants (promoter region and 5′ or 3′ untranslated regions), which were reported to cause disease in association with PHPT. The sequence reads of each sample were mapped to the human reference genome (GRCh37/hg19). The Burrows–Wheeler Aligner software was used for reading alignment. Duplicate read marking, local realignment around indels, base quality score recalibration, and variant calling were performed using the genomic analysis toolkit algorithms (Sentieon) for nDNA. Variant data were annotated using a collection of tools (VcfAnno and VEP) with a variety of public variant databases, including, but not limited to, the Genome Aggregation Database control population cohorts (gnomAD), ClinVar, and the Human Gene Mutation Database (30).

The sequence variant analysis pipeline was validated in the Clinical Laboratory Improvement Amendments (CLIA) and College of American Pathologists (CAP)-accredited Blueprint Genetics diagnostic laboratory. The series of selected quality criteria included the variant call quality score, variant genomic location, sequence content, and integrative genomics viewer visual analysis. This algorithm was established based on the outcome of internal validation performed in the CLIA and CAP-accredited Blueprint Genetics diagnostic laboratory.

CNV analysis was performed bioinformatically from next-generation sequencing data using a bioinformatics pipeline; one component was a CNV kit and the other involved in-house developed proprietary technology. CNVs were confirmed by digital polymerase chain reaction. The CNV analysis pipeline was validated in the CLIA and CAP-accredited Blueprint Genetics diagnostic laboratories. Sanger sequencing of the candidate variants was performed on the patient and family member samples to confirm the presence of the variant and the pattern of inheritance.

Variants were classified into five categories according to the American College of Medical Genetics and Genomics and the Association for Molecular Pathology (ACMG/AMP) guidelines (31): benign, likely benign, variant of uncertain significance (VUS), likely pathogenic (LP), and pathogenic (P). We reported all the variants classified as P, LP, or VUS. If any variant was found, genetic counselling of the proband and cascade genetic testing were offered to all at-risk family members. If required, loss of heterozygosity and CNV in tumours and immunohistochemistry studies were performed.

### Statistical analysis

Results corresponding to quantitative variables were presented as the mean ± standard deviation. Data associated with qualitative characteristics were reported as percentages. The normality of quantitative variables was assessed using the Shapiro-Wilk normality test. The significance of the difference between two means was assessed using Student’s T-test for normal variables and the Wilcoxon rank sum test for non-normal variables. The significance of the difference between more than two means was assessed using the Kruskal-Wallis test for non-normal variables. The significance of the difference between percentages was assessed using Pearson’s chi-square test. Differences were considered statistically significant at *P* < 0.05. Calculations were performed using R Commander version 4.0.5.

## Results

### General data

The clinical characteristics of the 40 participants (27 females and 13 males) are displayed in **Table 1**. The mean age at diagnosis of primary hyperparathyroidism was 51 ± 15.8 years. The circumstances leading to diagnosis of PHPT were diverse: 30% incidental findings of hypercalcemia, 20% symptomatic renal lithiasis, 17.5% palpable cervical nodes, 17.5% neuromuscular symptoms, and other clinical features in the remaining patients. Thirty-two patients (80%) underwent surgery, with mean highest PTH levels of 50.28 (12.32-41.99) pmol/L (RV 1.06-6.89), mean albumin adjusted serum calcium of 3.03 ± 0.5 mmol/L, and mean nadir phosphate levels of 0.84 ± 0.39 mmol/L. The inclusion criteria were as follows: 15 subjects were under 45 years of age at diagnosis of PHPT; 28 cases met the histology criteria (one parathyroid carcinoma, 11 atypical parathyroid adenomas, six cystic parathyroid adenomas, and five MGD); four patients presented with recurrence of PHPT after surgery; two cases were included because of family history of PHPT; and seven patients presented tumours that could be related to familial syndrome. 32% of the patients met two or more inclusion criteria.

**Table 1:** Clinical variables of the 40 participants.

Clinical variables of the cohort (n = 40)			
Mean age at diagnosis of hyperparathyroidism (years)	51 ±15.8		
Gender: female/male, n (%)	27 (68%) / 13 (32%)		
Clinical presentation	Incidental finding of hypercalcemia		n = 12 (30%)
	Renal lithiasis		n = 8 (20%)
	Palpable cervical node		n = 7 (17.5%)
	Neuromuscular symptoms		n = 7 (17.5%)
	Other signs or symptoms (osteoporosis, fractures, etc.)		n = 6 (15%)
Patients who underwent surgery, n (%)	n = 32 (80%)		
Mean PTH max pre-surgery (pmol/L)	50.28 (12.32-41.99)		
Mean adjusted Ca max pre-surgery (mmol/L)	3.03 ±0.5		
Mean P min pre-surgery (mmol/L)	0.84 ±0.39		
Inclusion criteria (indication for genetic testing)	Classification of participants into categories according to inclusion criteria		Absolute values*
	Age ≤45 years old	7 (18%)	15 (37.5%)
	Parathyroid carcinoma	0 (0%)	1 (2.5%)
	Atypical parathyroid adenoma	6 (15%)	11 (27.5%)
	Cystic parathyroid adenoma	2 (5%)	6 (15%)
	Multiglandular disease	4 (10%)	10 (25%)
	Recurrent hyperparathyroidism three months after surgery	2 (5%)	4 (10%)
	Family history of primary hyperparathyroidism	2 (5%)	2 (5%)
	Associated-tumor suggestive of familial syndrome **	4 (10%)	7 (17.5%)
	≥2 inclusion criteria	13 (32%)	

Data are expressed as mean ± standard deviation or interquartile range. PTH max: highest serum parathyroid hormone levels (RV 1.06-6.89) before surgical treatment or during follow-up in non-operated patients; Ca: serum calcium levels; P: serum phosphate levels; SD: standard deviation; *Classification including patients with ≥2 inclusion criteria in the corresponding category; **Associated tumors suggestive of familial syndrome: prolactinoma (ID3), GH-producing adenoma (ID5 and ID19), clear cell renal carcinoma (ID14), lymphoblastic lymphoma (ID22), Aberrant T-cell population (ID29), metastatic duodenal neuroendocrine tumour, melanoma, and renal angiomyolipoma (ID33).

Genetic sequencing of the targeted panel identified five pathogenic/likely pathogenic germline variants in five of the 40 participants (three in *CASR* and two in *CDKN1B*) (12.5%) and four variants of uncertain significance (one in *CDKN2C*, one in *CASR*, and two in *TRPV6*) (10%). There was one frameshift variant, one nonsense variant, and seven missense variants. **Table 2** summarizes the genetic and clinical characteristics of patients with germline variants.

**Table 2:** Genetic and clinical characteristics of patients displaying germline variants in any of the genes included in the panel.

ID	Age	Sex	Gene	Variant	Variant type	ACMG	ACMG classification	Previously reported (gnomAD / Pubmed)	Ca Pre	Ca Post	PTH Pre	PTH Post	Cr	CCCR	Inclusion criteria*	Pathology	Gland weight (gr)	Identified carriers
18	47	M	*CDKN1B*	c.280_281delinsG, p.(Pro94Alafs*25)	Frameshift	LP	PVS1 (very strong), PM2 (supporting)	No / No	3.39	2.20	55.24	6.89	1.07	NA	3	APA	15.7	Mother (colon neoplasm)
29	34	M	*CDKN1B*	c.169C>T, p.(Gln57*)	Nonsense	P	PVS1 (very strong), PM2 (moderate)	No / No	3.37	2.15	379.1	6.25	2.20	0.010	9 (1, 3, 5, 8)	MGD: 3 parathyroid hyperplasia glands and 1 APA	1.7	None
27	28	F	*CDKN2C*	c.319T>G, p.(Leu107Val)	Missense	VUS	PM2 (moderate)	0.0036%/ No	2.84	1.97	9.43	4.55	0.90	0.030	9 (1, 4)	MGD: 1 cystic adenoma and 1 parathyroid adenoma	0.1	Mother (PHPT)
15	77	M	*CASR*	c.1172T>G, p. (Phe391Cys)	Missense	LP	PM2 (moderate), PP3 (supporting) PP2	No / No	4.12	1.68	212.93	3.39	1.07	0.005	9 (3, 4, 5)	MGD: 1 APA (with cystic cells) and 1 hyperplasia gland	1.33	Son (asymptomatic PHPT)
21	49	M	*CASR*	c.413C>T, p. (Thr138Met)	Missense	P	PM1 (moderate), PP2 (supporting), PM2 (moderate), PP3 (supporting), PP5 (strong)	No / Yes	3.12	-	15.37	-	0.80	0.004	7	-	NA	Daughter with symptomatic PHPT (not genetic test performed)
24	64	F	*CASR*	c.2393C>T, p. (Pro798Leu)	Missense	LP	PM1 (moderate), PP2 (supporting), PM2 (moderate), PP3 (supporting), PP5 (strong)	No / Yes	2.80	2.34	13.78	7.74	0.46	0.013	9 (5, 6)	MGD: 3 lipohyperplasia glands	0.28	2 sons and 1 daughter with asymptomatic PHPT
31	75	F	*CASR*	c.175G>A, p. (Glu59Lys)	Missense	VUS	PM1 (supporting), PM2 (supporting), BP4 (moderate)	No / No	2.45	2.33	12.08	8.69	1.02	0.011	5	MGD: 2 hyperplasia glands	0.08	2 sisters and 1 brother with mild PHPT (normal calcium)
4	41	M	*TRPV6*	c.1937G>A, p. (Arg646Gln)	Missense	VUS	PM2 (moderate)	0,0032%	3.30	2.43	15.90	3.71	0.91	0.011	1	Parathyroid adenoma	0.4	NA
30	35	F	*TRPV6*	c.787G>A, p. (Asp263Asn)	Missense	VUS	PM2 (moderate	0,0415%	2.75	-	15.58	-	0.62	0.010	1	-	-	NA

ID: identification number of each case; RV: reference value; Age: age at diagnosis of primary hyperparathyroidism; F: female; M: male; ACMG: variant classification according to ACMG guidelines; LP: likely pathogenic; P: pathogenic; VUS: variant of uncertain significance; Ca-Pre (mmol/L): corrected serum calcium pre-surgery (RV 2.1-2.55); Ca-Post (mmol/L): corrected serum calcium post-surgery; PTHPre (pmol/L): serum parathyroid hormone pre-surgery (RV 1.06-6.89); PTHPost: serum parathyroid hormone post-surgery; Cr: serum creatinine at diagnosis of primary hyperparathyroidism; CCCR: Calcium creatinine clearance ratio; APA: atypical parathyroid adenoma; MGD: multigland disease; Carriers: family members who carried the same variant and their clinical characteristics; PHPT: primary hyperparathyroidism; NA: not available; * Inclusion criteria (indication for genetic testing): 1: age ≤45 years old; 2: parathyroid carcinoma; 3: atypical parathyroid adenoma; 4: cystic parathyroid adenoma; 5: multiglandular disease; 6: recurrent hyperparathyroidism 3 months after surgery; 7: family history of primary hyperparathyroidism; 8: associated tumour suggestive of familial syndrome; 9: ≥2 inclusion criteria.

### *CASR* variants

Four patients carried heterozygous missense variants of the *CASR* gene (ID15, ID21, ID24, and ID31): two likely pathogenic, one pathogenic, and one VUS. Two of the *CASR* variant carriers had CCCR < 0.01, and the others were between 0.01-0.02. Of the four patients, three underwent surgical treatment.

**Figure 2:**
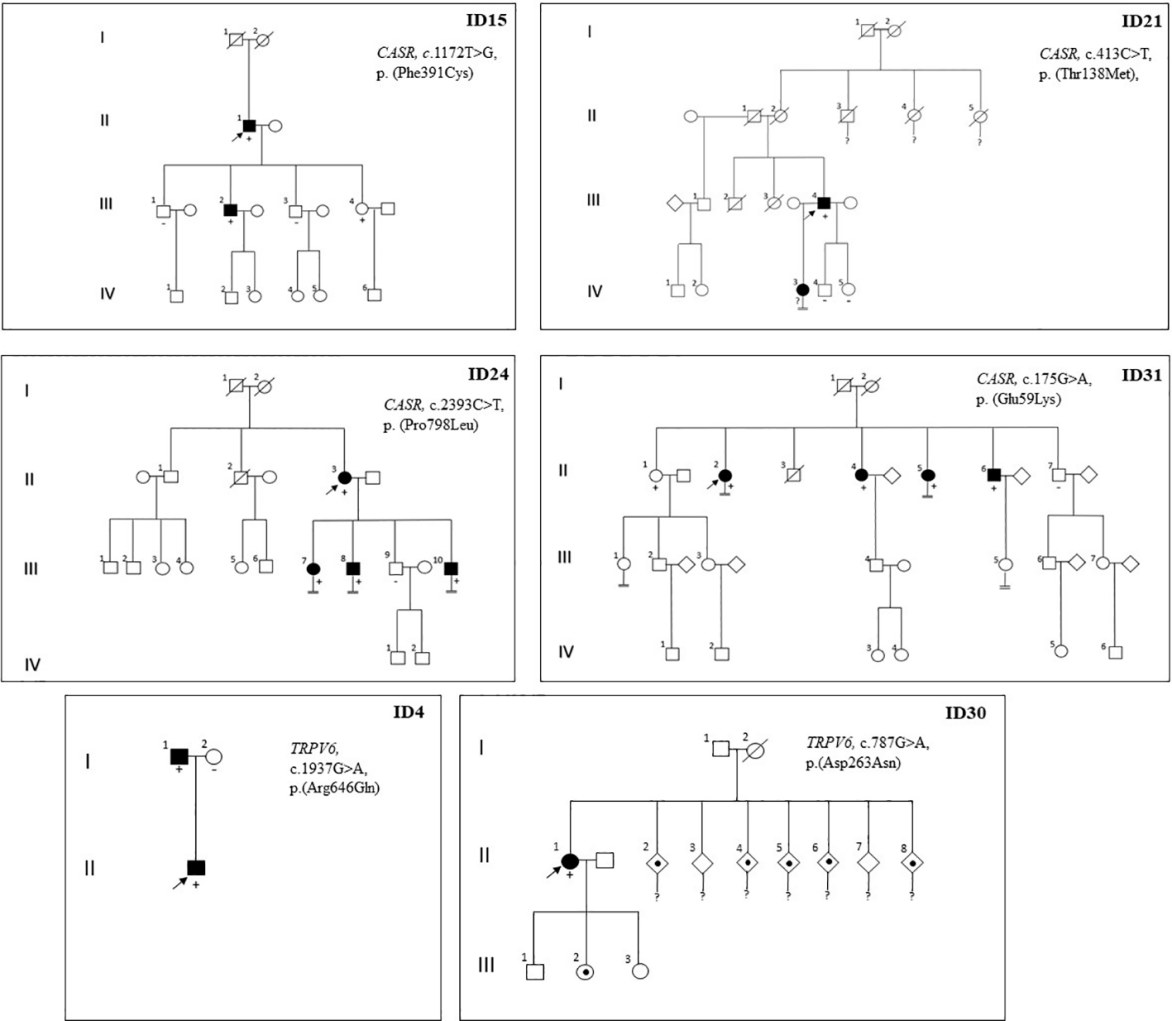
Pedigree of probands with detection of germline variant of *CASR* or *TRPV6* genes and their relatives. Family members are indicated by generation (Roman numerals) and individuals (Arabic numerals). Arrows indicate index case; Circle, female; Square, male; Double line below descent line, no offspring; Empty symbol, unaffected family member; Filled symbol, affected family member (inappropriately non-suppressed or high parathyroid hormone levels with normal or elevated serum calcium levels); Filled spot, history of nephrolithiasis or severe vitamin D deficiency; Slashed symbol, deceased; +, mutation carrier; -, non mutation carrier;?, no genetic test performed.

Proband ID15 presented with severe symptomatic hypercalcemia at the time of PHPT diagnosis that required hospital admission. Computed tomography of the neck revealed a partially cystic lesion posterior to the thyroid gland, suggesting cystic parathyroid adenoma. Surgery was performed, and the pathological diagnosis was MGD due to one atypical parathyroid adenoma with cystic cells and one hyperplastic parathyroid gland. After surgery, the patient remained normocalcemic and asymptomatic for four years of follow-up until now. A genetic test revealed c.1172T>G, p.(Phe391Cys) variant of *CASR*, classified as LP, according to ACMG. Biochemical tests detected hypercalcemia plus CCCR 0.01 in one of the two family members who carried the identical germline variant as the proband; conversely, no alterations in calcium or PTH serum levels were found on non-mutation carrier relatives (see family pedigree in **Figure 2**).

Cases ID24 and ID31 were diagnosed with PHPT in an osteoporosis study (defined by a T score below -2.5 SD). In both cases, the Sestamibi scan findings were consistent with a single parathyroid lesion. Even so, bilateral surgery was performed in both cases due to the observation of multiple enlarged glands during the procedure. Pathology study showed multiglandular disease in both cases (three lipohyperplasia glands in case ID24 and two hyperplasia glands in case ID31) (See **Table 2**). Densitometric osteoporosis improved after parathyroid excision in both cases, although patient ID24 showed persistent biochemical PHPT after surgery. Cascade genetic testing and blood tests were offered to all first-degree relatives. Biochemical PHPT (characterized by elevated or inadequately normal PTH levels with varying degrees of hypercalcemia) was detected in multiple family members who share the same variants as the probands (**Figure 2**). They were offered clinical and analytical follow-up, although to date none of them required parathyroid surgery. In the ID31 family, segregation was observed in all but one sibling. Yet, variant c.175G>A, p.(Glu59Lys) was classified as VUS for the time being, according to the ACMG criteria (PM1 (supporting), PM2 (supporting), and BP4 (moderate)). Case ID21 was included in the study because he had one daughter with PHPT. His imaging localization studies were negative, so surgery was not performed, but treatment with cinacalcet was initiated (30 mg once daily) and calcium plasma levels decreased from 3.12 to 2.74 mmol/L (RV 2.1-2.55). The proband’s daughter declined to undergo a genetic test.

### *CDKIs* variants

As our group recently reported, three novel *CDKIs* germline variants were identified in three unrelated cases: two likely pathogenic variants of *CDKN1B* and one VUS variant of *CDKN2C* (see **Table 2**) (11). Patients harbouring *CDKN1B* variants were included in the study because of atypical parathyroid adenomas, plus age under 45 years at diagnosis in case ID29. Case ID27, who carried the *CDKN2C* variant, was included due to having a young age at diagnosis and cystic parathyroid adenoma. Neoplasm screening showed other non-endocrine tumours in the three cases: single colon adenoma with dysplasia and atypical lipomas (defined as lipomatous neoplasms with focal cellular atypia) in case ID18, an aberrant T-cell population in case ID29, and a non-functional pituitary adenoma in case ID27. Familial segregation analysis of these variants identified the same variant of the *CDKN2C* gene in ID27’s mother, who was diagnosed with PHPT with moderate hypercalcemia and osteoporosis due to parathyroid adenoma. She underwent surgery and subsequently achieved normal calcium and PTH levels. Genetic analysis of tumour samples showed uniparental disomy of chromosome 1, including *CDKN2C*, in case ID27. Neither loss of heterozygosity nor CNV for the *CDKN1B* gene was found in cases ID18 and ID29, respectively. Clinical data are comprehensively described in the mentioned paper (11).

### *TRPV6* variants

We detected two variants of the *TRPV6* gene, classified as VUS, in two unrelated cases that were included in the study by virtue of having a young age at PHPT diagnosis (**Table 2**). Both probands had a CCCR of 0.01. Case ID4, who had a clinical history of nephrolithiasis, was diagnosed with PHPT at 41 years old. He underwent surgery for a single parathyroid adenoma; after surgery, his PTH and calcium plasma levels remained normal. Familial segregation analysis was performed on his parents (his only living first-degree relatives). His father carried the same genetic variant and was diagnosed with asymptomatic normocalcemic primary hyperparathyroidism through analytical tests. Case ID30 was diagnosed with PHPT and severe vitamin D deficiency after her newborn developed severe hypocalcaemia due to rickets. Vitamin D treatment was started for both the patient and the newborn. After normalizing vitamin D serum levels, PTH and calcium levels remained elevated in case ID30. Neck ultrasound and Sestamibi scan revealed a nodule suggestive of a parathyroid lesion within the thymus. The proband was advised to undergo surgery, but she failed to follow up, and the family segregation research could not be completed. The only information available about the first-degree relatives is that five of the seven siblings of the index case had a history of nephrolithiasis (see family pedigree in **Figure 2**).

### Clinical data according to genetic test results

We analysed the clinical and biochemical features of the study participants according to their genetic test results (see **Table 3**). The median age at PHPT diagnosis of subjects with a positive genetic test result was not significantly different from that of patients with negative genetic results. Mean PTH and calcium serum levels pre-surgery of patients carrying genetic variants were higher than those with a negative genetic study, but the difference was not statistically significant. Serum phosphate levels were statistically similar between both groups. The only statistically significant difference found between the two groups was the CCCR, which was lower in the group of patients with a positive genetic test (0.01 vs 0.02, *P* = 0.0038).

**Table 3:** Characteristics of patients based on genetic test outcomes.

	Subjects with positive genetic test (n = 5)	Subjects with negative genetic test (n = 35)	*P*-value
Age at diagnosis (years)	54.2 ± 16	50.8 ± 15	0.6526
Calcium (mmol/L)	3.36 ± 0.49	2.99 ± 0.49	0.0817
PTH (pmol/L)	134.95 ± 159.25	38.18 ± 48.65	0.1582
Mean P min pre-surgery (mmol/L)	0.63 ± 0.19	0.87 ± 0.4	0.1005
Calcium creatinine clearance ratio (CCCR)*	0.0085	0.022	0.0057
More than one inclusion criteria (%)	3/5 (60 %)	10/35 (28.6 %)	0.1605

Data are expressed as mean ± standard deviation. The positive genetic test group included patients with LP or P variants, according to the ACMG criteria. The negative genetic test group included subjects with VUS variants or those without any variants found in the genes studied. *CCCR was analysed with Wilcoxon test being lower in subjects with class LP or P variants when compared to subjects with negative genetic test (P = 0.005). When analysing the differences in CCCR between patients who presented with each type of mutation (CASR, CDKN1B, or non-mutated; Kruskal-Wallis test), a statistically significant difference was observed (P = 0.02), mainly at the expense of subjects with mutated CASR, in whom the CCCR value was much lower.

## Discussion

In this study, we provide a well-defined cohort of 40 patients who presented features indicative of hereditary hyperparathyroidism. As far as we know, this is the largest cohort of patients with suspected FHPT reported in Spain where an expanded panel of genes related to FHPT was systematically studied. We identified nine germline variants (two of *CDKN1B*, one of *CDKN2C*, four of *CASR*, and two of *TRPV6*) in nine participants (22.5%): five pathogenic or likely pathogenic variants (12.5%) and four VUSs (10%). The most common confirmed genetic disorder in our sample was FHH type 1 (due to *CASR* variants), followed by MEN4 (due to *CDKN1B* variants). Notably, seven of these variants have not been previously reported (**Table 2**).

The prevalence of germline variants found in our study is consistent with some previous studies conducted in individuals with clinical diagnoses of FHPT and is slightly higher than other cohorts. In the Japanese population, *CDC73*, *MEN1*, and *CASR* were studied in 13 families with suspected FHPT due to variable risk phenotypes, resulting in four variants (30%) (32). Conversely, only one *MEN1* mutation was found in all the genes studied in a cohort of 29 Finnish patients with suspected FHPT (3%) (28). Other studies in different susceptible populations were conducted, resulting in a variable prevalence of germline variants: 8/86 (9.3%) in the American cohort (29), 9/62 (15%) in South Australia (7), and 18/68 (26%) in New Zealand (7). One of the largest studies to date identified 19 pathogenic germline variants (11 *CASR*, 6 *MEN1*, 1 *CDC73*, and 1 *AP2S1*) in 121 British patients referred for genetic testing for suspected FHPT (16%) (33). However, all these results are hardly comparable, as the authors used different risk criteria for FHPT and the genes assessed differed between the studies (7, 21, 22, 27, 33, 34).

In our study, the inclusion criteria were based on the established features of inherited tumour susceptibility. The histological diagnosis of the surgical pathology specimens included in this study was based on the outdated WHO criteria. However, it is important to note that the nomenclature has since been updated. The concept parathyroid hyperplasia is no longer generally supported in the context of PHPT as it consists of multiple “clonal” neoplastic proliferations. The 2022 WHO classification approves PHPT-related multiglandular parathyroid disease as germline susceptibility-driven neoplasm. Also, previously referred to as atypical parathyroid adenomas are now recognized as atypical parathyroid tumours (35). As shown in **Table 3**, in a comparison of different characteristics of the participants according to the genetic results, the only statistically significant difference was in CCCR. There were no statistically significant differences in the mean age of the group carrying LP or P variants compared to that of subjects with a negative genetic study (54.2 vs. 50.8 years, respectively). Calcium and PTH concentrations were somewhat higher in the group of patients with a positive genetic test but without statistically significant differences from the group with a negative test.

Interestingly, subjects carrying LP or P variants had a lower CCCR than those with a negative genetic study (Wilcoxon test, *P* = 0.057). When we analysed the differences in CCCR between patients who presented with each type of mutation (*CASR*, *CDKN1B*, or non-mutated; Kruskal-Wallis test), a statistically significant difference was observed (*P* = 0.02) but mainly at the expense of subjects with mutated *CASR*, in whom the CCCR value was much lower (see **Table 3**). Of note, a CCCR of <0.01 is generally considered a well-planned screening method of FHH. However, this cut-off is of limited clinical value due to reduced diagnostic sensitivity (only captures ~65% of FHH type 1 patients) and reduced specificity (~18% of surgically confirmed PHPT cases have CCCR <0.01). Also, CCCR is not validated for diagnosing FHH in patients with renal impairment, vitamin D insufficiency, or pregnancy (36).

Apart from CCCR, we did not find a trait that was clearly more frequent in patients in whom we found germline variants. Although the differences were not statistically significant, the group of patients carrying a genetic variant had a greater tendency to present more than one inclusion criterion, i.e., various risk factors (60% vs. 28.6% in patients with a negative genetic study). This suggests that having more than one risk characteristic may increase the possibility of positive genetic screening. However, it should be noted that our sample was small and that this is a descriptive study, so it is difficult to draw firm conclusions. Studies with a larger number of patients would be necessary to corroborate this hypothesis.

As mentioned above, four subjects were diagnosed with FHH type 1 due to *CASR* variants. Variants c.413C>T, p.(Thr138Met) and c.2393C>T, p.(Pro798Leu) were earlier reported in patients with a diagnosis of FHH (37, 38). Instead, variants c.1172T>G, p.(Phe391Cys) and c.175G>A, p.(Glu59Lys) were not previously described. Among the three patients who underwent surgery, one achieved a cure, while the remaining two did not, which is typically observed in cases of FHH. However, both of these cases showed improvement in osteoporosis following the surgery. This supports the notion that parathyroid surgery might be beneficial and even curative in some cases of FHH. Of note, the c.175G>A, p.(Glu59Lys) variant was not documented in gnomID, a reference population database. Therefore, this variant was classified as a VUS according to ACMG criteria (**Table 2**). Even so, we believed it was appropriate to report this variant since the clinical features of the index case and most of the carrier relatives were consistent with FHH type 1. As noted in previous publications, FHH is a rare autosomal dominant disorder with low or variable penetrance, which could be the reason for the incomplete segregation of the disease in the ID31 family (39).

Historically, FHH and PHPT have been classified as distinct conditions owing to some differences, such as lower urinary calcium excretion (typically CCCR ≤0.01), generally a more benign clinical course, and the controversial role of parathyroid surgery in FHH patients, as parathyroid hyperplasia of more than one gland is present in the majority of cases. However, recent research has shown that FHH can be classified as a subtype of FHPT because they share a similar pathophysiology. The *CASR* gene is known to play a role in parathyroid growth; therefore, we suppose that some variants may promote both hyperplasia and parathyroid adenoma formation (33). There are several reported cases of parathyroid adenoma in patients with FHH (33, 40, 41). To our knowledge, we report for the first time a case of atypical parathyroid tumour in a patient with FHH type 1 (ID15). In FHH type 1, CASR hypoactivity leads to increased renal calcium reabsorption and increased PTH production, which can result in hypercalcemia with inappropriately normal or elevated PTH. Although traditionally considered rare, 40% of FHH cases could present with atypical features like renal and bone damage, hypercalciuria, severe hypercalcemia, CCCR>0.02, or parathyroid adenoma (42). In agreement with this, of the four *CASR* variant carriers we reported, one had severe hypercalcemia requiring hospitalization (ID15) and three had bone damage (osteoporosis). The presence of osteoporosis more strongly suggests the acquisition of autonomy of one or more parathyroid glands.

A prevalence of 7.5% (3/40) of variants in *CDKIs* was found in our group. Two novel likely pathogenic *CDKN1B* variants were found in patients with atypical parathyroid adenomas (**Table 2**). *CDKN1B* mutations are responsible for MEN4 syndrome, which causes a MEN1-like phenotype with other related tumours (11, 43). Additionally, a novel variant of the *CDKN2C* gene with uniparental disomy was classified as VUS in a patient with PHPT and a non-functional pituitary adenoma. To our knowledge, *CDKI*s variants have not been reported in patients with atypical or cystic adenomas. As variants in *CDKIs* have been shown to be a potential cause of FHPT, which may also give rise to other tumours, we suggest that these genes should be added to the FHPT study gene panels. Despite the potential significance of germline variants of these genes, the reported incidence among affected patients is limited (11, 43). Further research is required to determine the actual prevalence of these mutations and the extent of their phenotypic impact.

We report two variants of uncertain significance in *TRPV6* in two patients with PHPT diagnosed before the age of 45 years (ID4 and ID30). *TRPV6* encodes the TRPV6 channel, which plays a vital role in intestinal and renal calcium absorption. TRPV6 protein is expressed in many epithelial tissues, including the intestine, kidney, placenta, epididymis, and exocrine glands such as the pancreas, prostate, salivary glands, sweat, and mammary glands. Transient severe neonatal hyperparathyroidism has been reported in patients with missense variants of *TRPV6*, inherited in an autosomal recessive manner. The disease is characterized by transplacental maternal-foetal calcium transport interference, causing a foetal calcium deficit, resulting in neonatal hyperparathyroidism and insufficient bone mineralization (44, 45).

Variants in the *TRPV6* gene have also been associated with nephrolithiasis and chronic pancreatitis when inherited in an autosomal recessive manner. However, most studies investigating the genotypic and phenotypic effects of *TRPV6* were conducted in mice (45, 46). In the present study, two cases (ID4 and ID30) were diagnosed with primary hyperparathyroidism in young adulthood and carried a single *TRPV6* variant. To our knowledge, there have been no reports of adults with dominant inheritance of *TRPV6* involvement that presented with PHPT. Thus, we report these variants to expand the understanding of this gene. However, their clinical significance remains unclear and should be interpreted with caution. Further research is necessary to determine the clinical implications of *TRPV6* variants inherited in a dominant manner. A potential strategy could be to perform whole exome sequencing (WES) or whole genome sequencing (WGS) to study patients with inconclusive genetic test results. This approach may help to uncover a second variant that was not detected by a gene panel and may reveal novel genes associated with FHPT.

We did not find any variants in the remaining genes included in the panel. One point of note is the absence of *MEN1* in the studied cohort. The average percentage of *MEN1* pathogenic variants in families with clinical FIHP is around 18% (26). This absence in our cohort could be attributed to the exclusion of individuals previously known to carry *MEN1* mutations. In our hospital, *MEN1* gene analysis is frequently performed in clinical practice rather than in other genes (47). It should be noted that previous studies associated atypical parathyroid tumours with mutations in *CDC73* (48). In contrast, variants in *CDC73* were not found in 11 patients with these tumours included in our cohort.

This study has several limitations. The exclusion of cases of FHPT with known mutations in any of the studied genes might have distorted the actual prevalence of germline variants in the at-risk population, which could be higher than reported. Also, there may be a selection bias as: some patients may not express risk features because a variable clinical expressivity and/or different penetrance of the characteristics associated with each of the various syndromic forms of PHPT at the time of PHPT diagnosis, etc (49). Another limitation was the small number of participants, which made it difficult to extract statistically significant results applicable to the population. It should be noted that four of the variants found in our cohort were classified as VUS, so their clinical relevance should be interpreted with caution. However, this classification system is not absolute and is dependent on the accuracy of the available evidence at the time of assessment (50). In addition, we did not conduct functional analysis; therefore, we could not confirm whether the variants caused the characteristics observed in the patients, particularly in the case of those classified as VUS. On the other hand, the genetic testing we conducted was limited to gene panel sequencing.

In summary, we found a remarkable prevalence of genetic variants among the genes studied in the at-risk population. This is consistent with the hypothesis that FPHP is probably an underdiagnosed entity owing to non-standardized genetic screening. In our cohort, genetic testing allowed the early diagnosis and treatment of PHPT and other related comorbidities of probands and their first-degree relatives. We suggest genetic testing in patients with PHPT who present any of the risk characteristics considered in the inclusion criteria of our study. WES strategy is replacing gene panel studies in clinical laboratories. Thus, we propose a filtering procedure using the aforementioned customized gene panel updated as a first-tier virtual panel for patients with suspected FHPT.

## Data availability statement

The datasets presented in this study can be found in online repositories. The names of the repository/repositories and accession number(s) can be found in the article/supplementary material.

## Ethics statement

Written informed consent was obtained from all patients, and the study was approved by the institutional Ethics Committee of Institut d’Investigació i Innovació Parc Taulí I3PT. The studies were conducted in accordance with the local legislation and institutional requirements. The participants provided their written informed consent to participate in this study. Written informed consent was obtained from the individual(s) for the publication of any potentially identifiable images or data included in this article.

## Author contributions

IC and IM-A designed the study, analysed and interpreted the data, and wrote, reviewed, and edited the manuscript. NB performed and interpreted genetic data and wrote and edited the manuscript. MB-C, SB, XG, and RP participated in editing the manuscript. AC and MR provided scientific guidance. All authors approved the final version of the manuscript to be published.
